# Protection from Ultraviolet Radiation during Childhood: The Parental Perspective in Bavaria

**DOI:** 10.3390/ijerph13101011

**Published:** 2016-10-14

**Authors:** Olaf Gefeller, Wolfgang Uter, Annette B. Pfahlberg

**Affiliations:** Department of Medical Informatics, Biometry and Epidemiology, Friedrich Alexander University of Erlangen-Nuremberg, Erlangen D-91054, Germany; wolfgang.uter@fau.de (W.U.); annette.pfahlberg@fau.de (A.B.P.)

**Keywords:** children, skin cancer, primary prevention, sun protection, ultraviolet radiation

## Abstract

During childhood, parents play a vital role in sun protection of their children. Their guidance is essential for avoiding excessive exposure to ultraviolet (UV) radiation, a risk factor for developing skin cancer in later life. In a population-based cross-sectional study conducted between October 2011 and February 2012, we assessed how 3281 parents implemented sun protection for their three- to six-year-old children in practice. In particular, clothing, shade-seeking behavior, wearing of sunhats and sunglasses, use of sunscreens and the amount of time spent outdoors were ascertained in two settings (beach, garden/playground). The results showed that the overall level of parental sun protection for their children in the beach setting, and to a lesser extent also in the everyday outdoor setting, is relatively high. Using sunscreens with a high sun protection factor and instructing children to wear a sunhat were very common. Lesser attention was paid to sun-protective clothing, seeking the shade and wearing sunglasses. The amount of time spent outdoors during summer days was high. Therefore, the recommendation to completely avoid sun exposure during peak UV times around noon during summertime needs to be reinforced. In addition, the observed difference in the protective behavior between the beach and an everyday outdoor setting points to the necessity to encourage better sun protection for children also in outdoor activities of daily living.

## 1. Introduction

For several decades, the incidences of malignant melanoma and non-melanoma skin cancer have been rising in all countries with fair-skinned populations including Germany [1,2,3,4]. As a consequence, skin cancer has become a major public health topic nowadays [5]. Ultraviolet (UV) radiation plays an important role in the pathogenesis of various forms of skin cancer and has been established as their main environmental risk factor by numerous epidemiological and laboratory investigations [6,7]. Not only adults are partly heavily exposed to solar and artificial UV radiation, but also children are often subject to high solar UV exposure during outdoor activities. About 25%–50% of the lifetime UV exposure apparently occurs before 18–21 years of age [8,9].

During childhood, parents play a vital role in sun protection of their children. They serve as a role model and their educational guidance is essential for behavior-forming of their children, including sun protection [10,11,12]. Educational campaigns for increasing public awareness of skin cancer risks and providing information on sun protective behavior have therefore focused on parents as a target group of special interest and importance [13]. In Germany, the Association of Dermatological Prevention (ADP), financially supported by Germany’s leading non-governmental funding and non-profit-making organization of oncology-related activities (German Cancer Aid), has set the agenda for educational campaigns during the last 25 years. The first nationwide campaign targeting specifically parents of young children was launched under the slogan “child and sun” in 1993 [14] and has been repeated several times in the following decades [15]. Regional and local skin prevention activities have supplemented the ADP activities. Of particular importance for Bavaria were efforts by the Bavarian Cancer Association, for example, the SunPass project to improve sun protection of children by implementing a certification system for interested kindergartens [16]. However, the mix of preventive activities at different levels did not follow a coordinated strategy.

The current practice of sun protection during childhood has to be assessed in population-based surveys to be able to monitor potential progress and to identify remaining areas for further improvement. For methodological reasons, such cross-sectional studies—even when performed repetitively over time—cannot prove that the educational campaigns had any effect on sun protective behavior as they have only an observational character and thus limited ability to identify causes in a methodologically strict sense. Here, we describe results from a large Bavarian population-based cross-sectional study among parents with children aged three to six years old concerning their parental sun-protective activities. In particular, we address age-, sex-, region- and setting-specific differences in sun-protective guidance, thereby providing detailed insights into the variation of parental sun protection in practice.

## 2. Materials and Methods

### 2.1. Study Participants

The ErlKing-II Study (Erlangen Kindergarden study; the suffix II has been used to distinguish this study from an earlier study [17] in Erlangen) was performed during the winter of 2011/2012 (starting at the end of October 2011 and ending mid-February 2012) in the Bavarian districts of Erlangen and Ansbach after approval of the study by the local ethics committee at the University of Erlangen-Nuremberg. This region in Southern Germany comprises an urban area (the city of Erlangen with 110,000 inhabitants), a rural part (the county of Erlangen with 133,000 inhabitants) and the compactly settled non-urban area of Ansbach (a town of 40,000 inhabitants). Using official administrative information, we contacted all 93 kindergartens in the district of Erlangen supervising more than 50 children and all 19 kindergartens in the district of Ansbach. Of these, 98 (87.5%) consented to participate in the survey, allowing us to contact the parents of all children enrolled in these kindergartens. Overall, 5812 questionnaires were distributed to the parents via the kindergarten teachers. If a family had two or more children attending the kindergarten, only one questionnaire was distributed to the parents, and information about the oldest child was requested. Hence, the sampling unit of the study was the household and all information referred to children from different families. Altogether 3281 (56.5%) questionnaires were returned, 3108 as paper-based questionnaires and 173 (5.4%) electronically via a secure web interface using an individualized password system to ensure that exclusively parents having received the invitation letter could enter data only once. Prior to the analysis, 152 had to be excluded because the children’s age was outside the pre-specified targeted age range. Finally, data on 3129 children from age three to six years old were included in the analysis.

### 2.2. Questionnaires

The self-administered standardized questionnaire contained demographic characteristics, such as the child’s age and gender, maternal and paternal age, and photosensitivity data, such as the child’s hair and iris color. The majority of questions addressed how parents implemented sun protection for their children during the summer of 2011, the summer preceding the survey. In particular, they were asked regarding precautionary measures taken when their children played outside on a summer day in two different settings, namely, in the garden or the playground as an everyday setting and during beach holidays. Six aspects relevant for sun protection were ascertained: (i) type of clothes worn (predominantly naked, swimsuit, T-shirt/shorts, long-sleeved clothes, clothes with special UV protection); (ii) frequency of staying in the shade (rarely/never, occasionally, mostly, always); (iii) wearing a sunhat (yes/no); (iv) wearing sunglasses (yes/no); (v) frequency of using sunscreens (rarely/never, once a day, every 2–3 h); and (vi) level of the sun protection factor (SPF) for sunscreen users (<30, 30–49, ≥50). In addition, the amount of time the child typically spent outdoors during a summer day between 10 a.m. and 5 p.m. was assessed.

### 2.3. Statistical Analysis

Statistical analysis was performed using SAS 9.4 (SAS Institute Inc., Cary, NC, USA). Descriptive information on the frequencies and percentages (relative to the total sample size or the sample size of the corresponding subgroup, respectively) observed in this survey has been tabulated in five tables using an identical systematic structure. Heterogeneity of the response patterns between subgroups defined by sex (male/female children), age of the children (3, 4, 5, 6), age of the mother (≤30, 31–35, 36–39, ≥40) and region (urban/Erlangen, rural/Erlangen-Höchstadt, small-town area/Ansbach) were evaluated using different statistical tests depending on the type of the outcome variable and the structure of the variable defining the subgroups. When dichotomous outcome variables (sunhat, sunglasses) were analyzed, we applied the χ^2^-test to address sex- and region-specific differences and the Cochran–Armitage trend test to examine the relation to the age of children and mothers, respectively. When outcome variables with an ordinal structure (clothing, shade-seeking behavior, sunscreen use, level of SPF) were evaluated we used the Mann–Whitney test for comparing male and female children, the Kruskall–Wallis test for region-specific comparisons and the Jonckheere–Terpstra trend test for age-related comparisons. The length of the children’s outdoor stay was analyzed by the same nonparametric statistical tests as in the case of ordinal outcome variables due to the distributional properties of this outcome. In all analyses, addressing discrepancies between subgroups defined by maternal age only questionnaires filled out with maternal involvement were analyzed, thereby excluding 213 questionnaires. *p*-values less than 0.05 were considered statistically significant.

## 3. Results

Of the 3129 questionnaires, 91.4% were completed by the mother alone, 6.3% by the father alone, and 2.0% by both parents and the small remainder by other household members. The average age of mothers and fathers were 35.8 ± 5.3 (mean ± standard deviation) and 38.9 ± 6.1 years, respectively. The average age of the children was 4.4 ± 1.0 year. Of the 3129 questionnaires, 1532 referred to girls and 1579 to boys; 18 families did not specify the gender of their children. There was virtually no difference between the age distributions of girls and boys.

### 3.1. Clothing

Parents’ answers regarding the different types of clothing typically worn by their children in a beach setting during summer holidays and in an everyday summer setting at the playground or in the garden are shown in Table 1. Pronounced discrepancies between the two settings are apparent. Whereas children wore predominantly T-shirts and shorts at the playground and in the garden, most children wore swimsuits at the beach reflecting the difference in outdoor activities of children in the two settings. Interestingly, more than 20% of children are protected by special clothing with an integrated UV protection in the beach setting, but only 2% of the children use this type of UV clothing in the everyday setting.

When looking at the subgroup-specific results, a strong heterogeneity of results depending on children’s age is evident in both settings. On the one hand, younger children wore clothing with special UV protection more often than older children, but younger children, on the other hand, also played predominantly naked more often than older ones. Thus, it is difficult to interpret this finding as showing better protection of younger children by the type of clothing. We also observed significant differences in the children’s clothing depending on their mother’s age. Children with older mothers used special clothing with UV protection and long-sleeved clothes to a larger extent than children with younger mothers.

### 3.2. Shade-Seeking Behavior

Table 2 provides information about shade-seeking behavior in the beach and garden setting, respectively. More than 70% of the parents stated that they instruct their children to look for shade mostly or always in the beach setting. This figure dropped slightly to 64% in the everyday setting. Strong discrepancies between younger and older children were found, with younger children instructed more often to look for shade than older children in both settings. Depending on the age of the mother, we also observed differences in the distribution of shade-seeking behavior in the beach setting. Younger mothers paid more attention to this sun protective measure than older mothers.

### 3.3. Sunglasses and Sunhats

Sunglasses were less used often than sunhats by the three- to six-year-old children in our survey (Table 3). Only one quarter of the children wore sunglasses in the garden or at the playground. This low proportion increased to 38% at the beach. Girls used sunglasses significantly more often than boys in both settings. Younger age of the mother was also strongly correlated with a higher proportion of children wearing sunglasses in both settings.

In contrast to the use of sunglasses, wearing sunhats was very common in the study group (Table 3). Ninety-four and 86% of the children, respectively, used sunhats in the beach and everyday settings, respectively. Younger children and children of younger mothers used sunhats significantly more often than older children and children of older mothers, respectively. In the beach setting, these relations were less strong, but still noted, on a higher level of overall use of sunhats.

### 3.4. Sunscreen Use

Sunscreen was widely used by the parents to protect their children from UV radiation in both settings. Table 4 reports the different frequencies of use in the beach and everyday setting. In the beach setting, 82% of the parents answered that they used sunscreens for their children every 2–3 h. Virtually no one used sunscreens rarely or never in this setting. In the everyday setting, in the garden, or at the playground, the majority of parents used sunscreens for their children once a day. Similar to what we observed for wearing sunglasses and sunhats, we found that younger children and children of younger mother used sunscreens more often than older children and children of older mothers, respectively.

Information on the distribution of the SPF of the sunscreens applied by the parents is given in Table 5. The majority of sunscreens have a very high SPF of 50 or above. Sunscreens with a SPF lower than 30 were applied by less than 10% of the parents at the beach and by 16% in the everyday setting. Children’s and mothers’ ages were strongly related to the preference of high SPF sunscreens. In both settings, younger mothers used high SPF sunscreens significantly more often than older mothers and younger children were more often protected by high SPF sunscreens than older children.

### 3.5. Duration of Outdoor Stay

In addition to the assessment of precautionary measures taken by the parents to protect their children when staying outdoors, we also ascertained the average duration of such outdoor stays during summer days between 10 a.m. and 5 p.m. when children are supervised by the parents. Parents stated that their children stayed outdoors on average 3.9 h (±1.1) h during the seven-hour-long period. There was no sex-specific difference with respect to the length of the period. However, we observed a gradual increase in the duration of the outdoor stay from three-year old to six-year old children. The mean duration increased significantly (*p* = 0.002) by 6.4% (or in absolute terms: 15 min) from the youngest to the oldest age group. Maternal age and duration of outdoor stay showed the opposite relationship: younger mothers reported a significantly (*p* = 0.002) higher duration of outdoor stay of their children than older mothers. The mean length of the outdoor stay decreased gradually by 16 min (6.4%) from the youngest to the oldest maternal age group.

Considering the relationship between the different sun-protective measures and the duration of outdoor stay, we observed that the mean length of an outdoor stay increased in children less protected by clothing and shade, respectively, in both settings. For example, children wearing long-sleeved clothes in the garden or at the playground had a shorter mean outdoor stay by 47 min (18.4%) than predominantly naked children or children with swimsuits. No relationships between the duration of the outdoor stay and sunscreen use as well as wearing sunglasses or sunhats were apparent.

### 3.6. Regional Differences

When describing the subgroup-specific results for the different sun-protective measures in the five previous subsections, the comparison between the three study regions had purposely not been mentioned. In this subsection, we summarize the findings of regional comparisons. For most sun-protective measures (Table 1, Table 2, Table 3, Table 4 and Table 5), we observed only small differences between the three study regions. This also held true for the comparison of the length of outdoor stay, where the mean duration was nearly the same in all three regions (Ansbach: 3.8 ± 1.1, Erlangen: 3.9 ± 1.1, Erlangen-Höchstadt: 3.9 ± 1.1; *p* = 0.16).

The only regional difference in the beach setting was observed for the type of clothing, where parents of children in Ansbach used special clothing with UV protection less often than in the other two regions. Parents in the small town of Ansbach differed from the parents in the two other regions also in the everyday setting with respect to their instructions regarding shade-seeking behavior, use of sunscreens and wearing of sunhats and sunglasses. Children from Ansbach were told to seek shade, use sunscreens, wear sunhats and sunglasses more often than children in the other two study regions.

## 4. Discussion

Based on a large population-based survey in different Bavarian districts, we provided a detailed description of the current practice of sun protection of three- to six-year-old children in different outdoor settings. Our data enable a comparison of actual sun-protective behavior of parents with children aged 3–6 years with the advice given in skin cancer awareness and prevention campaigns, thereby mirroring what has been achieved by these educational activities so far. Overall, the level of parental sun protection for their young children in the beach setting and to a lesser extent also in the everyday outdoor setting is high; however, a closer look at the single sun-protective measures taken by the parents revealed some prevailing deficits.

Whereas almost all parents tried to protect their children by using sunscreens with a high SPF and by instructing them to wear a sunhat, lesser attention was given to sun-protective clothing, seeking the shade frequently and wearing sunglasses. In particular, the importance of wearing sunglasses was considerably underrated by the parents. Another deficit of the practical implementation of sun-protective messages given in all educational campaigns addressing skin cancer prevention related to the recommendation to avoid sun exposure during peak UV times around noon. In our survey, the average length of an outdoor stay between 10 a.m. and 5 p.m. was nearly four h with more than 28% of the children staying outdoors five h or longer. This means that a substantial proportion of children did not avoid sun exposure during peak UV times in the summertime. Admittedly, we ascertained only the length of outdoor stay during a seven-hour-period covering a wider time interval than the two-hour-period from 12 a.m. to 2 p.m. that is explicitly mentioned as the peak UV time around noon in most skin cancer prevention campaigns. Thus, we cannot directly estimate the proportion of children being exposed to UV radiation around noon. Still, we can indirectly infer from the distribution of the duration of outdoor stay that a non-negligible proportion of children had to be exposed at peak UV time. Their parents evidently did not follow the preventive recommendations so far.

Previous studies investigated sun-protection behavior of children during beach holidays [18,19,20,21] or in everyday circumstances [22], but only one previous study compared sun-protection activities between these settings [23]. In this German study from the beginning of the century, the data showed that—except for clothing habits—parents chose more frequently a higher level of sun protection for their children on the beach than in an everyday outdoor setting. A similar picture of results emerged in our current study, with the exception that nowadays a substantial proportion of children, i.e., every fifth child, is protected by special UV clothing in the beach setting, which is a modern development. This difference in protective behavior between the two settings reveals a misunderstanding about the risk of UV exposure. Apparently, a substantial proportion of parents think that UV radiation on the beach is intense and harmful, while it is mild and harmless in an everyday outdoor setting. Thus, the necessity to adopt protective measures for their children also in everyday situations during summertime needs to be stressed.

In our subgroup comparisons, we identified the age of the children and of the mothers as being particularly important for the implementation of sun-protective measures, while the gender of the children and the region of residence played a minor role. The relationship between children’s age and sun protection has been discussed in several earlier studies [24,25,26,27]. Our study confirmed that even for young children within the narrow age range of three to six years, the level of sun protection was reduced with increasing age. Whether this trend is a consequence of a reduction of parental efforts to protect their older children or of self-determined actions of older children paying less attention to parental guidance cannot be answered by our study. The impact of maternal age on sun-protective activities cannot be summarized in a single sentence, and the results showed a more complex picture. On the one hand, younger mothers used sunscreens more often and—when using sunscreens—applied those products with a very high SPF more often than older mothers. They also instructed their children to wear sunglasses and sunhats more frequently than older mothers. On the other hand, older women protected their children better in the beach setting with respect to their clothing as seen in the remarkably higher proportion of children with older mothers wearing special clothing with UV protection. It can only speculated whether the observed discrepancies reported by mothers of different age groups reflect age-specific preferences of appearance (sunglasses, sunhats), age-related financial opportunities to spend more money for expensive clothing with UV protection or age-dependent differences in the adoption of preventive measures due to informed decisions how to protect their children. Our study data cannot be used to distinguish between these different explanations.

Further limitations of our investigation relate to the pervasive problem of self-selection in population-based surveys and the problematic validity of self-administered questionnaires employed in such surveys. It can never be ruled out that the group of non-responders would have answered differently than the responding parents. Although our self-administered questionnaire had been used in three earlier surveys in other German regions [28,29,30,31,32] and was extensively pretested during the preparation of the current study, we cannot be sure that all parents understood the questions as intended. In addition, in current surveys among parents on sun protection for their children, the potential of some special form of information bias has to be taken into account. Parents may have overreported their sun protective guidance and behavior to reflect socially desirable behavior [33,34] instead of giving accurate information about their actual sun-protective activities. Such information bias will lead to an overoptimistic assessment of the current level of sun protection achieved for young children and needs to be evaluated in validation studies comparing self-reported sun-protective behavior and objectively observed UV exposure and protective measures [35]. Recall and memory bias have also to be taken into account as potential problems in our study as parents were asked about behavior and preventive activities during the preceding summer preceding that dated back several months at the time when filling out the questionnaire. The magnitude of inaccuracy in reporting about the last summer has, however, been found to be low in a US study [36].

## 5. Conclusions

Our study provided detailed population-based data on the current practice of parental sun-protective guidance of young children in Bavaria. Although the results demonstrated that the overall level of sun protection achieved is relatively high, several deficits became apparent. Most importantly, the recommendation to completely avoid sun exposure during peak UV times around noon during summertime needs to be reinforced. In addition, the observed difference in the protective behavior between the beach and an everyday outdoor setting points to the necessity to encourage better sun protection for children also during outdoor activities of daily living.

## Figures and Tables

**Table 1:** Types of clothes worn in two outdoor settings (beach—upper part and garden—lower part) based on answers of 3129 parents of three- to six-year old children in the Erlking-II-study in three Bavarian districts in Southern Germany.

Setting/Subgroup			Size of Group (*n*)	Type of Clothing					*p*
				Naked (%)	Swimsuit (%)	T-Shirt/Shorts (%)	Long Sleeves (%)	Ultraviolet Clothing (%)	
Beach Setting	Sex of Children	Male	1579	3.4	46.8	27.3	1.2	21.4	0.030
		Female	1532	3.4	52.7	23.3	1.4	19.3	
	Age of Children	3	716	5.7	42.4	23.6	2.1	26.3	<0.001
		4	958	2.9	48.4	25.4	1.2	22.1	
		5	1031	3.0	54.0	26.5	0.9	15.7	
		6	402	1.4	55.2	24.5	0.8	18.1	
	Age of Mother	≤30	473	2.5	56.9	23.5	0.7	16.4	<0.001
		31–35	887	4.6	50.4	23.7	1.0	20.3	
		36–39	845	3.3	47.1	26.9	1.1	21.7	
		≥40	700	2.2	46.3	25.3	2.2	24.1	
	Region	Erlangen	1121	3.0	50.2	24.9	1.6	20.3	<0.001
		Ansbach	476	3.3	56.6	25.9	0.7	13.4	
		Erlangen-Höchstadt	1532	3.7	47.2	25.3	1.2	22.6	
Garden Setting	Sex of Children	Male	1579	1.8	4.4	89.1	2.2	2.4	0.489
		Female	1532	1.7	5.7	88.2	2.0	2.4	
	Age of Children	3	716	3.0	4.6	85.2	3.2	4.1	0.126
		4	958	1.4	5.5	89.0	1.5	2.6	
		5	1031	1.6	5.0	90.1	2.2	1.3	
		6	402	1.2	5.5	89.8	1.5	2.0	
	Age of Mother	≤30	473	2.1	7.2	86.9	1.1	2.8	0.003
		31–35	887	2.1	4.7	89.3	1.2	2.6	
		36–39	845	1.6	4.8	89.4	2.0	2.3	
		≥40	700	1.6	3.9	88.7	3.3	2.6	
	Region:	Erlangen	1121	1.8	4.9	89.1	2.4	1.8	0.657
		Ansbach	476	1.5	7.0	87.1	1.3	3.2	
		Erlangen-Höchstadt	1532	1.8	4.6	88.8	2.1	2.7	

**Table 2:** Shade-seeking behavior in two outdoor settings (beach—upper part and garden—lower part) based on answers of 3129 parents of three- to six-year old children in the Erlking-II-study in three Bavarian districts in Southern Germany.

Setting/Subgroup			Size of Group (*n*)	Frequency of Seeking Shade				*p*
				Rarely/Never (%)	Occasionally (%)	Mostly (%)	Always (%)	
Beach Setting	Sex of Children	Male	1579	5.64	24.6	50.6	19.2	0.068
		Female	1532	4.2	23.2	52.2	20.4	
	Age of Children	3	716	3.2	21.4	52.8	22.6	0.004
		4	958	5.4	23.1	52.5	19.0	
		5	1031	5.8	25.6	48.8	19.7	
		6	402	4.5	25.6	52.5	17.4	
	Age of Mother	≤30	473	3.9	21.9	48.7	25.6	0.027
		31–35	887	4.5	22.8	53.0	19.7	
		36–39	845	4.9	24.1	52.2	18.9	
		≥40	700	5.1	24.1	52.1	18.7	
	Region	Erlangen	1121	5.7	25.2	49.5	19.6	0.212
		Ansbach	476	4.3	22.5	51.9	21.3	
		Erlangen-Höchstadt	1532	4.5	23.4	52.6	19.5	
Garden Setting	Sex of Children	Male	1579	6.9	30.7	52.5	9.9	0.024
		Female	1532	6.0	28.6	53.2	12.2	
	Age of Children	3	716	4.6	24.3	59.0	12.0	<0.001
		4	958	7.4	29.5	52.5	10.7	
		5	1031	7.2	32.3	48.7	11.8	
		6	402	6.0	32.2	53.1	8.7	
	Age of Mother	≤30	473	3.4	26.6	56.8	13.2	0.093
		31–35	887	7.3	28.4	52.8	11.6	
		36–39	845	6.3	33.3	50.6	9.9	
		≥40	700	6.3	27.4	55.1	11.2	
	Region	Erlangen	1121	8.6	30.8	49.7	10.9	<0.001
		Ansbach	476	4.4	24.1	58.1	13.3	
		Erlangen-Höchstadt	1532	5.6	30.5	53.4	10.5	

**Table 3:** Relative frequencies of wearing sunglasses and sunhats in two outdoor settings (beach—upper part and garden—lower part) based on answers of 3129 parents of three- to six-year old children in the Erlking-II-study in three Bavarian districts in Southern Germany.

Setting/Subgroup			Size of Group (*n*)	Use of Sunglasses and Sunhats			
				Sunglasses (%)	*p*	Sunhat (%)	*p*
Beach Setting	Sex of Children	Male	1579	34.9	<0.001	93.9	0.739
		Female	1532	41.2		94.2	
	Age of Children	3	716	38.5	0.451	95.7	0.017
		4	958	39.0		94.7	
		5	1031	37.2		92.5	
		6	402	36.9		93.5	
	Age of Mother	≤30	473	48.4	<0.001	95.1	0.051
		31–35	887	37.5		95.2	
		36–39	845	35.9		94.0	
		≥40	700	34.1		92.9	
	Region	Erlangen	1121	35.4	0.006	93.0	0.230
		Ansbach	476	44.3		94.3	
		Erlangen-Höchstadt	1532	38.1		94.6	
Garden Setting	Sex of Children	Male	1579	22.6	<0.001	87.5	0.006
		Female	1532	27.8		84.0	
	Age of Children	3	716	28.1	0.029	91.2	<0.001
		4	958	24.9		85.0	
		5	1031	25.1		83.1	
		6	402	21.5		84.8	
	Age of Mother	≤30	473	38.4	<0.001	89.9	<0.001
		31–35	887	26.9		88.2	
		36–39	845	18.3		85.7	
		≥40	700	21.7		81.5	
	Region	Erlangen	1121	23.3	0.003	83.2	0.003
		Ansbach	476	31.3		89.2	
		Erlangen-Höchstadt	1532	24.7		86.5	

**Table 4:** Frequency of using sunscreens in two outdoor settings (beach—upper part and garden—lower part) based on answers of 3129 parents of three- to six-year old children in the Erlking-II-study in three Bavarian districts in Southern Germany.

Setting/Subgroup			Size of Group (*n*)	Frequency of Use			
				Rarely/Never (%)	Once a Day (%)	Every 2–3 h (%)	*p*
Beach Setting	Sex of Children	Male	1579	0.6	16.9	82.5	0.719
		Female	1532	0.7	17.3	82.0	
	Age of Children	3	716	0.1	14.7	85.2	0.007
		4	958	0.9	15.4	83.7	
		5	1031	1.1	19.8	79.1	
		6	402	0.0	18.0	82.0	
	Age of Mother	≤30	473	0.5	14.4	85.1	0.064
		31–35	887	1.2	15.0	83.8	
		36–39	845	0.3	19.0	80.7	
		≥40	700	0.8	17.7	81.6	
	Region	Erlangen	1121	0.4	18.2	81.5	0.585
		Ansbach	476	0.5	15.6	83.9	
		Erlangen-Höchstadt	1532	0.9	16.6	82.5	
Garden Setting	Sex of Children	Male	1579	6.7	54.5	38.8	0.046
		Female	1532	5.3	53.2	41.6	
	Age of Children	3	716	3.8	49.8	46.4	<0.001
		4	958	6.1	53.9	40.0	
		5	1031	7.6	55.7	36.7	
		6	402	5.6	54.0	40.4	
	Age of Mother	≤30	473	5.6	48.5	45.9	<0.001
		31–35	887	7.0	49.1	43.9	
		36–39	845	3.2	58.6	38.1	
		≥40	700	7.0	56.4	36.6	
	Region	Erlangen	1121	7.3	55.2	37.6	0.013
		Ansbach	476	7.0	48.0	45.0	
		Erlangen-Höchstadt	1532	4.7	54.2	41.0	

**Table 5:** Level of sun protection factor (SPF) when using sunscreens in two outdoor settings (beach —upper part and garden—lower part) based on answers of 3129 parents of three- to six-year old children in the Erlking-II-study in three Bavarian districts in Southern Germany.

Setting/Subgroup			Size of Group (*n*)	Level of SPF			*p*
				<30 (%)	30–49 (%)	≥50 (%)	
Beach Setting	Sex of Children	Male	1579	9.9	31.3	58.8	0.979
		Female	1532	9.7	31.9	58.5	
	Age of Children	3	716	8.4	28.4	63.3	0.003
		4	958	8.1	33.2	58.7	
		5	1031	11.9	31.3	56.8	
		6	402	10.9	33.2	55.9	
	Age of mother	≤30	473	7.9	26.4	65.7	<0.001
		31–35	887	7.4	30.5	62.1	
		36–39	845	10.2	31.4	58.5	
		≥40	700	12.8	33.1	54.0	
	Region	Erlangen	1121	10.3	30.8	58.9	0.592
		Ansbach	476	9.7	28.6	61.7	
		Erlangen-Höchstadt	1532	9.4	32.9	57.6	
Garden Setting	Sex of Children	Male	1579	16.2	37.4	46.4	0.720
		Female	1532	15.8	37.2	46.9	
	Age of Children	3	716	12.6	34.2	53.2	<0.001
		4	958	14.8	38.3	46.9	
		5	1031	19.1	36.8	44.1	
		6	402	17.0	41.0	42.0	
	Age of Mother	≤30	473	12.2	32.2	55.6	<0.001
		31–35	887	12.0	36.9	51.0	
		36–39	845	15.0	38.9	46.0	
		≥40	700	22.2	37.6	40.2	
	Region	Erlangen	1121	17.6	37.3	45.1	0.074
		Ansbach	476	14.9	33.5	51.7	
		Erlangen-Höchstadt	1532	15.3	38.4	46.3	
